# 

**Published:** 1879-01

**Authors:** 


### THE BUFFALO

### MEDICAL AND SURGICAL

### JOURNAL.

VOL. XVIII. JANUARY, 1879. NO. 6.

